# Matrin-3 is essential for fibroblast growth factor 2-dependent maintenance of neural stem cells

**DOI:** 10.1038/s41598-018-31597-x

**Published:** 2018-09-07

**Authors:** Kanako Niimori-Kita, Nobuaki Tamamaki, Daikai Koizumi, Daisuke Niimori

**Affiliations:** 1 Department of Molecular Pathology, Graduate School of Medical Sciences, Kumamoto University, 1-1-1, Honjo, Kumamoto, 860-8556 Japan; 2 Department of Morphological Neural Science, Graduate School of Medical Sciences, Kumamoto University, 1-1-1, Honjo, Kumamoto, 860-8556 Japan; 3 Department of Dermatology and Plastic Surgery, Graduate School of Medical Sciences, Kumamoto University, 1-1-1, Honjo, Kumamoto, 860-8556 Japan

## Abstract

To investigate the mechanisms underlying the maintenance of neural stem cells, we performed two-dimensional fluorescence-difference gel electrophoresis (2D-DIGE) targeting the nuclear phosphorylated proteins. Nuclear phosphorylated protein Matrin-3 was identified in neural stem cells (NSCs) after stimulation using fibroblast growth factor 2 (FGF2). Matrin-3 was expressed in the mouse embryonic subventricular and ventricular zones. Small interfering RNA (siRNA)-mediated knockdown of Matrin-3 caused neuronal differentiation of NSCs *in vitro*, and altered the cerebral layer structure of foetal brain *in vivo*. Transfection of Matrin-3 plasmids in which the serine 208 residue was point-mutated to alanine (Ser208Ala mutant Matrin3) and inhibition of Ataxia telangiectasia mutated kinase (ATM kinase), which phosphorylates Matrin-3 Ser208 residue, caused neuronal differentiation and decreased the proliferation of neurosphere-forming stem cells. Thus, our proteomic approach revealed that Matrin-3 phosphorylation was essential for FGF2-dependent maintenance of NSCs *in vitro* and *in vivo*.

## Introduction

Neural stem cells (NSCs) are self-renewing multipotent cells that generate the main phenotypes of the nervous system^1,2^. Mammalian NSCs differentiate into three major cell types—neurons, astrocytes, and oligodendrocytes—during development that involves multiple signal transduction pathways. The pathways include fibroblast growth factor (FGF) signalling, where a characteristic combination of transcription factors determines the fate of NSCs^3–5^. However, the molecular mechanism of the decision regarding the fate of NSCs is largely unknown.

Most related studies have tried to find molecules controlling NSC proliferation and differentiation^6,7^. The cDNA microarray is effective for the global analysis of the NSC differentiation state^8–11^, but is insufficient to detect protein quantity and post-translational modifications of critical nuclear factors, such as transcription factors, which have roles in the maintenance and differentiation of NSCs^12–14^. Proteomic analyses are more useful for studying protein quantity and post-translational modifications^15^. Therefore, we adopted the proteomic approach to elucidate signal switching of the NSC fate decision, and attempted to identify the nuclear factors with post-translational modifications that control signal switching of differentiation and the undifferentiated state of NSCs using the FGF stimulation model^16^. To identify the critical nuclear factors, we performed two-dimensional fluorescence-difference gel electrophoresis (2D-DIGE) targeting the nuclear phosphorylated proteins. Using 2D-DIGE, the critical nuclear factors and the phosphorylations that control NSC differentiation were detected. These included the phosphorylation of Matrin-3 in response to FGF2 stimulation.

To investigate the significance of Matrin-3 in NSC differentiation, Matrin-3 was analysed with a biochemical and cellular biological strategy combined with 2D-Western blotting, use of small interfering RNA (siRNA) *in vitro* and *in vivo*, treatment with Ataxia telangiectasia mutated (ATM) kinase inhibitor, over-expression of a phospho-mutant of Matrin-3 and immunohistochemical analysis. The collective results demonstrate the necessity for Matrin-3 phosphorylation to maintain NSCs.

## Results

### Matrin-3 targeting nuclear phosphorylated proteins is responsible for induction of NSCs by FGF2

**Figure 1:**
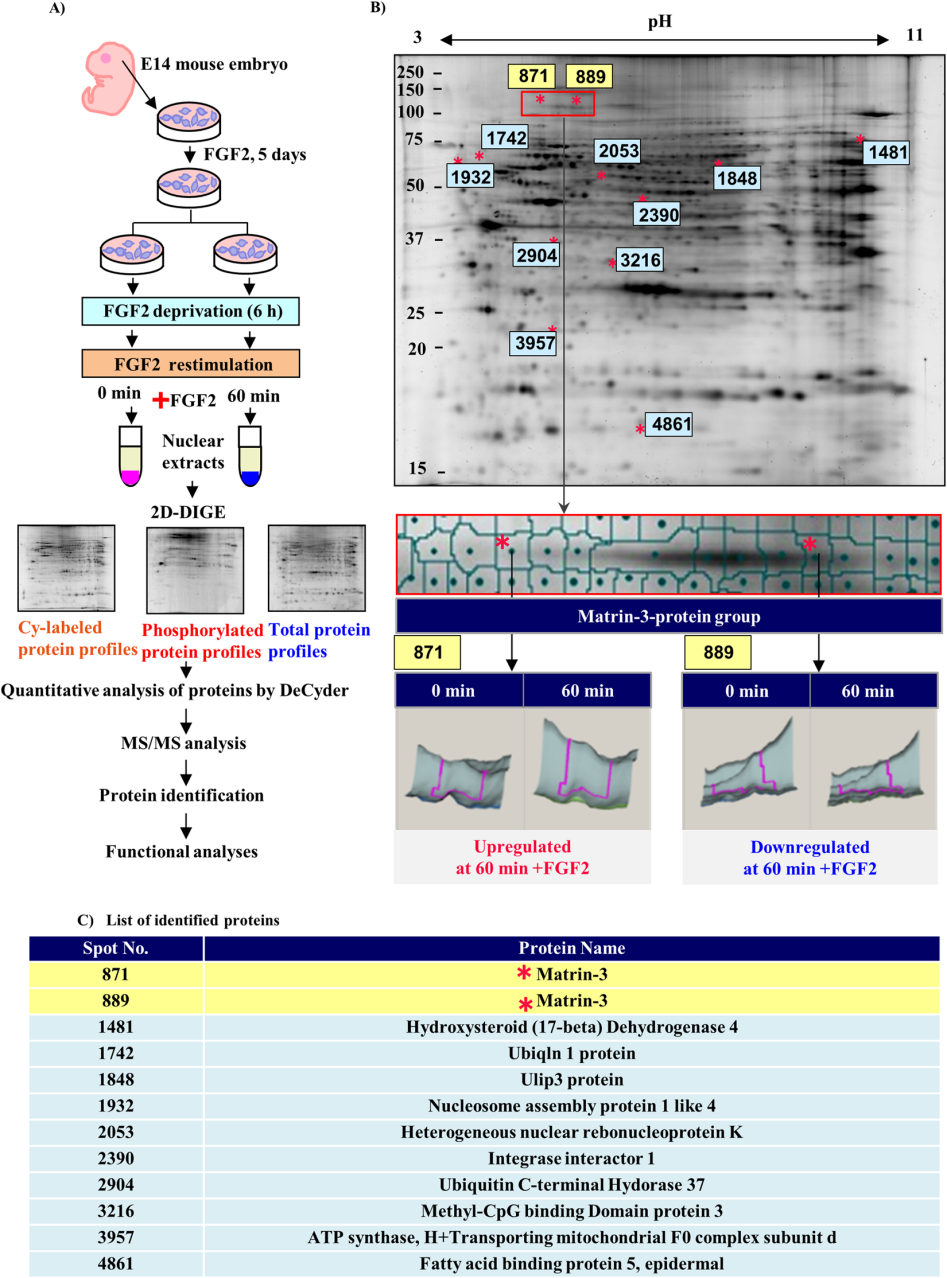
Identification of NSC maintaining phosphorylated proteins by 2D-DIGE and mass spectrometry analysis. (**A**) The experimental strategy for 2D-DIGE. (**B**) The 2D-DIGE map and 3D-images of up- or downregulated proteins. The asterisks indicate the up- or downregulated phosphoproteins. The red frame indicates the Matrin-3 protein group. Bottom panels (spots 871 and 889): 3D-images of Matrin-3 protein expression at 0 and 60 min after FGF2 stimulation following 6-h FGF2 deprivation. (**C**) The identified major proteins are listed. The spot numbers correspond to the annotation shown in (**B**). Detailed results of the protein identification are shown in Table S1.

We performed a global analysis of phosphorylated and non-phosphorylated nuclear proteins to detect the critical nuclear factors that control NSC differentiation (Fig. S1). Furthermore, we performed 2D-DIGE targeting the nuclear phosphorylated proteins with the FGF2 stimulation model (Fig. 1A). One pair of nuclear extracts was prepared using the stimulation model (n = 2) and the FGF2 stimulation experiment was performed four times (n = 2 × 4). In addition, four specimens were prepared by mixing equal amounts of eight specimens in total as internal control. A total of 12 samples were analysed by the 2D-DIGE comparative quantitative analysis. A total of 4095 protein spots were detected from 12 scanned 2D-DIGE images. Of these, 80 proteins whose expression was significantly changed by FGF2 stimulation (p < 0.05 using one-way ANOVA) were identified. Thirty of the spots represented phosphoproteins, as they were stained with ProQ-Diamond. The 30 spots were excised and analysed.

Mass spectrometry (MS) analysis identified 11 proteins (Fig. 1B,C) that were reportedly involved in transcriptional regulation, nuclear translocation, or chromatin modifications (Table S1). We focused on Matrin-3, which was the nuclear protein that was most highly phosphorylated by FGF2 stimulation (Figs 1B and S2A). FGF2 stimulation resulted in the appearance of acidic Matrin-3 (spots 871, 872 and 876) and decreased the content of the alkaline form (spot 889) (Fig. 1B). In particular, spot 871, which corresponded to spot 3 in Fig. S2, exhibited the most significant difference (p = 0.015, Student’s T-test). ProQ Diamond phosphostaining determined that 10 spots of the Matrin-3 protein group matched the phosphorylated protein profiles (Fig. S2B).

**Figure 2:**
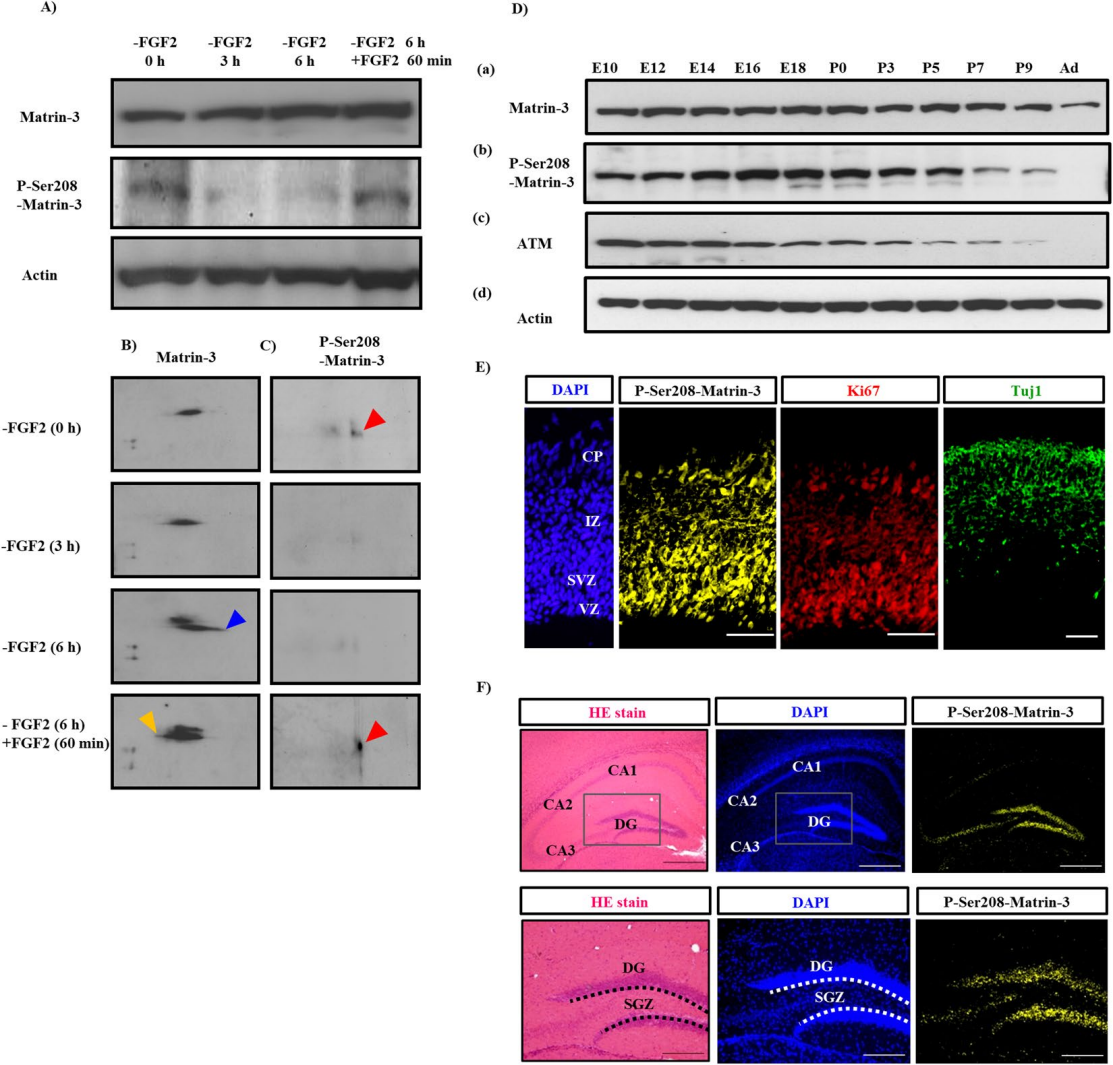
*In vitro* and *in vivo* expression of Matrin-3 and phospho-Matrin-3 (Ser208). (**A**) The expression of Matrin-3 and phospho-Matrin-3 (Ser208) in NSCs after FGF2 deprivation (-FGF2) and restimulation (+FGF2) on 1D-WB. (**B**,**C**) Mobility changes in phospho-Matrin-3 (P-Ser208-Matrin-3) and Matrin-3 after FGF2 deprivation and restimulation on 2D-WB. The lanes indicate the FGF2 stimulation times. (**B**) The blue arrowhead indicates the alkaline shift by dephosphorylation. The yellow arrowhead indicates the acidic shift by phosphorylation. (**C**) Red arrowheads indicate phospho-Matrin-3 (P-Ser208-Matrin-3) appearance. (**D**), (a) Expression of Matrin-3, (b) phospho-Matrin-3 (P-Ser208-Matrin-3), and (c) ATM from embrynic and adult mouse cerebra on 1D-WB. (**E**) Immunostaining of phospho-Matrin-3 (P-Ser208-Matrin-3), Ki67, and Tuj1 in the E14 mouse cortex (serial sections). CP; cortical plate, IZ; intermediate zone, SVZ; subventricular zone, VZ; ventricular zone. Actin is used as a control. Bar, 100 μm. (**F**) HE, DAPI staining and immunostaining of phospho-Matrin-3 (P-Ser208-Matrin-3) of the murine adult hippocampal dentate gyrus (same sections). DG; dentate gyrus, CA; cornet d’Ammon. Bars: 500 μm (upper panel), 200 μm (bottom panel); n = 5. Dotted line, subgranular zone (SGZ).

We did not observe a slight shift in the Matrin-3 band size after a 60-min stimulation with FGF2 following a 6-h deprivation (Fig. 2A), indicating that Matrin-3 phosphorylation was dependent on FGF2. Since it was difficult to detect Matrin-3 phosphorylation based only on molecular weight (Fig. 2A), we next performed 2D-western blot analysis (Fig. 2B). Since phosphoproteins shift to the acidic side in isoelectric focusing, 2D-western blot can advantageously detect the protein phosphorylation changes directly. The immunoreactivity of Matrin-3 at more acidic isoelectric pHs decreased after the 6-h FGF2 deprivation, while alkaline forms of Matrin-3 were enhanced and increased after 60-min FGF2 stimulation in the 2D-WB analysis (Fig. 2B). The shift detected by 2D-WB corresponded to the 2D-DIGE results.

### Matrin-3 is necessary to maintain NSCs

ATM phosphorylates Serine 208 (Ser208) of Matrin-3, and is involved in cell cycle modulation^17^. To confirm whether this residue of Matrin-3 is phosphorylated by FGF2 stimulation, 1D- and 2D-western blot analyses revealed phospho-Matrin-3 (P-Ser208-Matrin-3) at 0 h before FGF2 deprivation and at 60 min after FGF2 stimulation following a 6-h deprivation (Fig. 2A,C). The analyses also showed that Matrin-3 expression in the cortex persisted strongly until the adult stage (Fig. 2D-a), while phospho-Matrin-3 (P-Ser208-Matrin-3) appeared at the early embryonic stage, increased gradually, and then decreased after birth (Fig. 2D-b). ATM expression appeared slightly earlier than the peak of Matrin-3 phosphorylation (Fig. 2D-c). These results suggested that phospho-Matrin-3 (P-Ser208-Matrin-3) and ATM could be involved in NSC differentiation, mainly at the embryonic stage. Immunostaining of the E14 mouse cerebra revealed similar expression patterns of phospho-Matrin-3 (P-Ser208-Matrin-3) and Ki67 expression, but not Tuj1 (Fig. 2E). Moreover, phospho-Matrin-3 (P-Ser208-Matrin-3) was strongly expressed in mouse and human hippocampal dentate gyrus, including the subgranular zone, an area enriched in NSCs in adult neurogenesis (Figs 2F and S3)^18,19^. These results indicated that phospho-Matrin-3 at Ser208 could influence neural stem/progenitor cells during neural development and adult neurogenesis.

### Matrin-3-siRNA causes neuronal differentiation of NSCs *in vitro*, and changes the cerebral layer structure of the foetal brain after intraventricular injection

**Figure 3:**
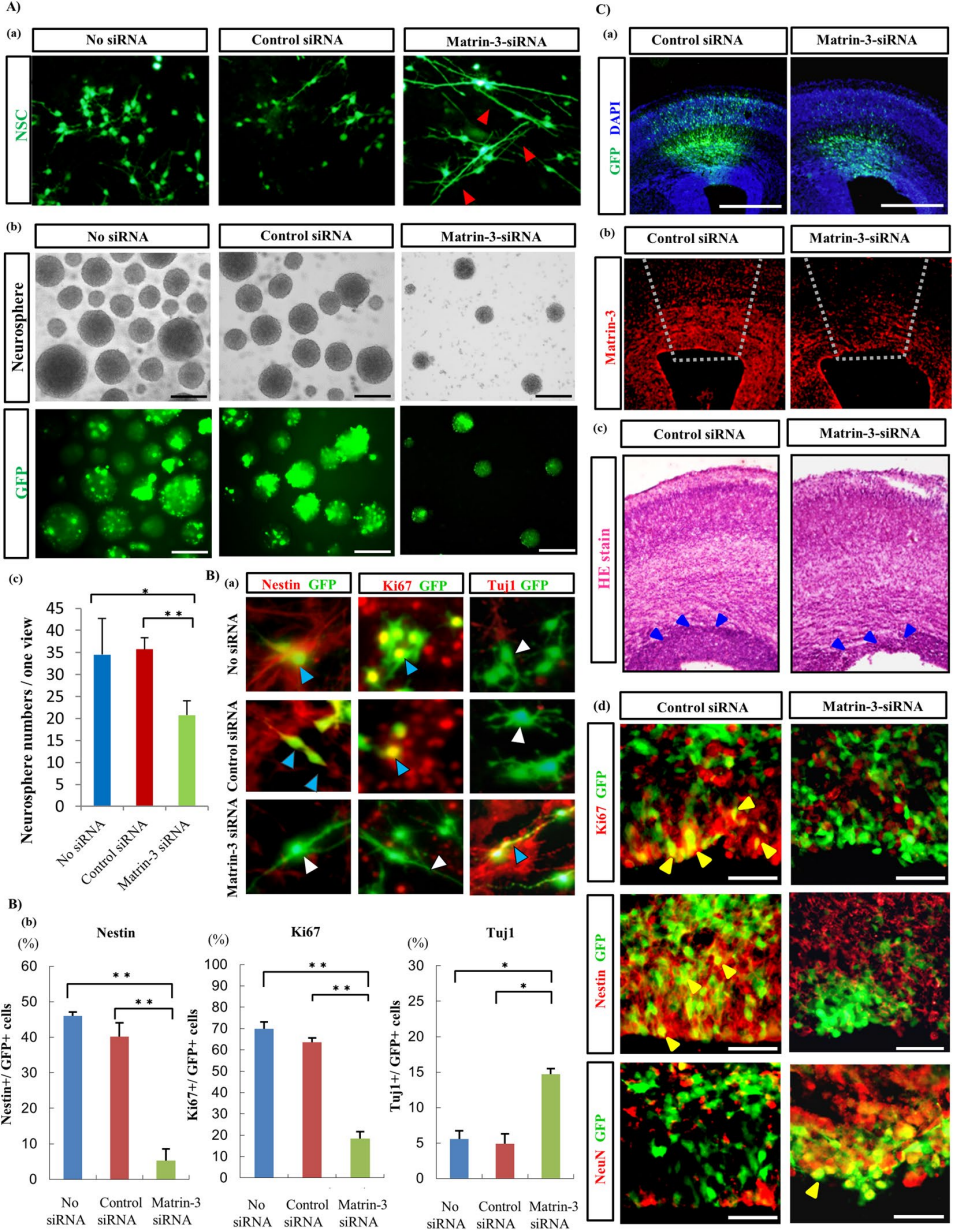
Significance of Matrin-3 for maintaining NSCs *in vitro* and *in vivo*. (**A**), (a) *In vitro* Matrin-3-siRNA induces extension of cellular processes of GFP^+^ NSCs. Red arrowheads indicate extension of cellular processes. (b) Matrin-3-siRNA reduced neurosphere-forming stem cells. Bar, 100 μm. (c) The number of GFP^+^ neurospheres per dish was counted. ^**^P < 0.01, ^*^P < 0.05 (one-way ANOVA plus Bonferroni/Dunn post-hoc test). Error bars, SE (5 separate experiments). (**B**), (a) Co-immunostaining for GFP and nestin, Ki67, or Tuj1 of NSCs *in vitro*. Cells are co-immunostained for GFP and nestin, Ki67 (blue arrowheads), or Tuj1 (white arrowheads). (b) Statistical measurements of neuronal differentiation in Matrin-3-knockdown cells. The number of nestin^+^, Ki67^+^ and Tuj1^+^ cells in GFP^+^ cells in one view was counted. ^**^P < 0.01, ^*^P < 0.05 (one-way ANOVA plus Bonferroni/Dunn post-hoc test). Error bars, SE (5 separate experiments). (**C**) In utero knockdown of Matrin-3 in the SVZ and VZ layer. (a) GFP^+^ cells in SVZ and VZ areas at E17.5. The cells are counterstained with DAPI. Bar, 500 μm. (b) Immunostaining of Matrin-3 shows the reduced expression in the Matrin-3 siRNA-treated tissue compared to the control siRNA-treated tissue. The dotted line surrounds the electroporated area. (c) HE staining revealed the disordered layer structure. Blue arrowheads indicate the SVZ/VZ area. (d) Co-immunostaining for GFP and nestin, Ki67, or NeuN in the VZ. Yellow arrowheads indicate nestin^+^, Ki67^+^ and NeuN^+^ cells in GFP^+^ cells. Bar, 50 μm; n = 4–5.

To confirm whether Matrin-3 can control NSC proliferation and differentiation, an experiment was performed using siRNA to knockdown the expression of Matrin-3. Matrin-3-siRNA and GFP gene co-transfected NSCs *in vitro* induced extension of their cellular processes (Figs 3A-a and S4). Additionally, the numbers of nestin^+^ and Ki67^+^ NSCs and nestin/Ki67 expression levels were reduced by Matrin-3-siRNA (Fig. 3B-a,b), while Tuj1^+^/GFP^+^ cells were increased (Fig. 3B-a,b). Therefore, siRNA-mediated knockdown of Matrin-3 promoted the extension of cellular processes and neuronal differentiation, accompanied by decreased cell proliferation. Matrin-3-siRNA did not induce glial differentiation or apoptosis (data not shown). Interestingly, Matrin3-siRNA reduced the number of neurospheres derived from NSCs (Fig. 3A-b,c). To determine whether Matrin-3 maintained the NSC system in the subventricular zone (SVZ) and ventricular zone (VZ), in utero electroporation of Matrin-3 siRNA into cortical VZ and SVZ cells was applied (Fig. 3C-b,d). Matrin-3-depleted cells remained in the SVZ and VZ (Fig. 3C-a), although control cells moved into the cortical plate and differentiated into neurons. Moreover, Matrin-3 depletion induced disordered SVZ and VZ layers (Fig. 3C-c), accompanying decreased Ki67+ and nestin+ cells and increased numbers of NeuN+ (neuronal marker) cells (Fig. 3C-d). Therefore, Matrin-3 could play a role in NSC maintenance *in vivo*.

### Transfection of Ser208Arg mutant Matrin-3 and inhibition of ATM kinase causes neuronal differentiation and decreases proliferation of neurosphere-forming stem cells

**Figure 4:**
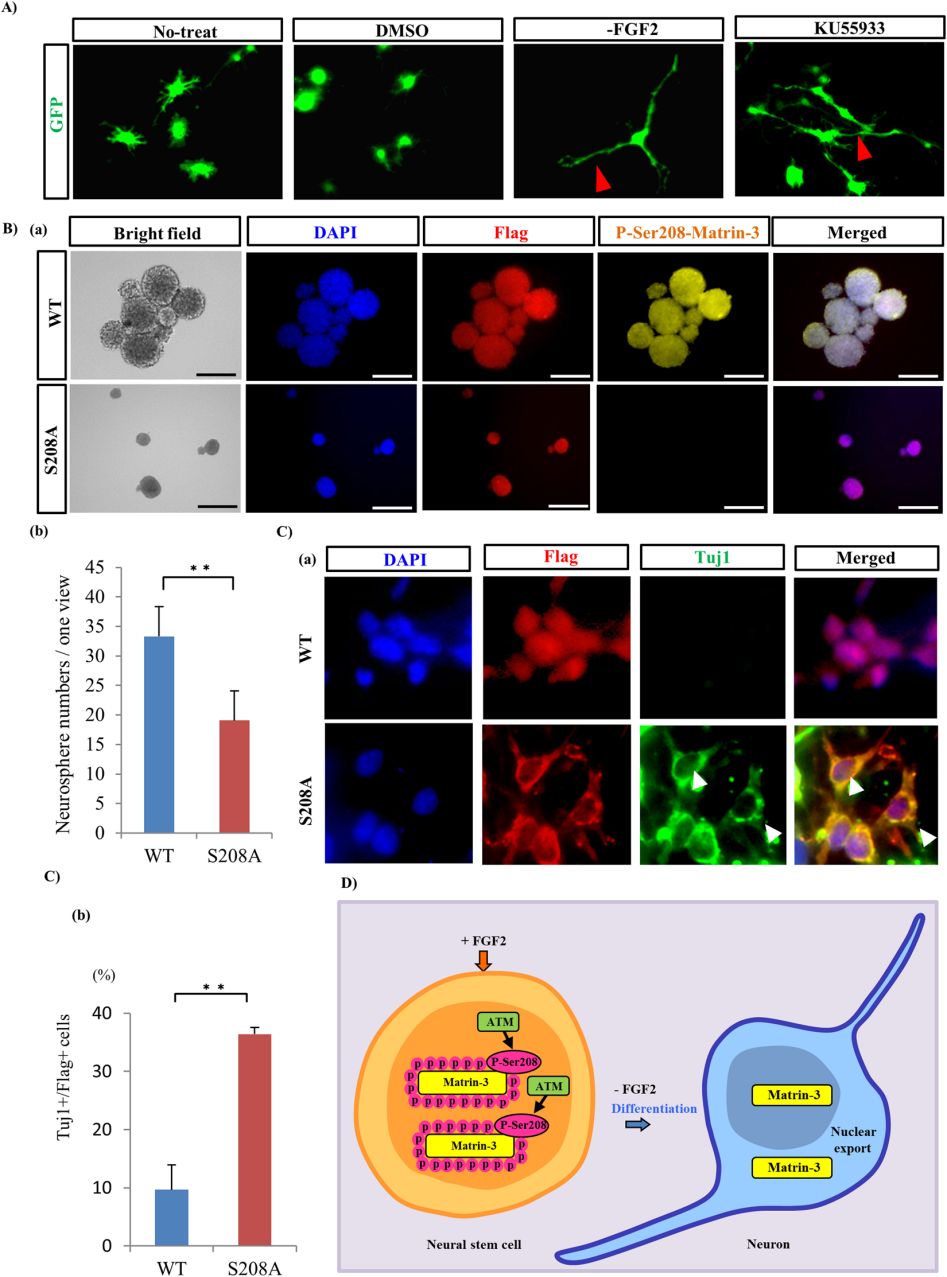
ATM phosphorylates Ser208 of Matrin-3 in the nucleus of NSCs. (**A**) Extension of cellular processes of NSC is induced by ATM inhibition (KU55933) *in vitro*. The FGF2 deprivation model (-FGF2) is used as an indicator of neuronal differentiation. Red arrowheads indicate extension of cellular processes. (**B**), (a) Phospho-mutant Matrin-3(Ser208Ala) inhibits the formation of neurospheres.P-Ser208-Matrin-3, Flag and DAPI images are merged. Bar, 100 μm. (b) Statistical measurements of Flag^+^ neurospheres in per dish were performed. ^**^P < 0.01 (Student’s t test). Error bars, SE (5 separate experiments). (**C)**, (a) Flag-Matrin-3 and Flag-Matrin-3-Ser208Ala plasmids were transfected into NSCs *in vitro*. Phospho-mutant Matrin-3 inhibits Matrin-3 nuclear localisation and induces neuronal differentiation. Flag, Tuj1, and DAPI images are merged. Tuj1^+^/Flag^+^ cells are observed to assess the nuclear translocation of Matrin-3 and neural differentiation. White arrowheads indicate incidence of Tuj1^+^ in Flag^+^ cells. (b) The bar graph indicates Tuj1^+^/Flag^+^ cells. ^**^P < 0.01 (Student’s t test). Error bars, SE (5 separate experiments). (**D**) Phosphorylation of Matrin-3 is necessary to regulate NSCs and to maintain self-renewal ability and the undifferentiated state.

Finally, to investigate whether phosphorylation of Matrin-3 at Ser208 is indispensable for maintaining NSCs, two additional experiments were done. One investigated the significance of ATM kinase for phosphorylation of Matrin-3 at Ser208. In the NSC differentiation experiment, western blotting and neurosphere assays in NSC cultures were performed using an ATM kinase inhibitor (KU55933). KU55933 suppressed the appearance of phospho-Matrin-3 at Ser208 in NSCs, induced extension of NSC processes (Figs 4A, S5 and S6) and reduced the number and size of neurospheres derived from NSCs (Fig. S7). The second experiment sought to confirm the functional significance of the Ser208 phosphorylation site. Flag-Matrin-3 and mutant plasmids containing point mutations of Ser208 to alanine (Ser208Ala) were transfected into NSCs (Fig. 4B,C). Neurosphere assays demonstrated reductions in the number and size of Flag-Matrin-3-Ser208Ala-expressing neurospheres compared to Flag-Matrin-3-expressing neurospheres (Fig. 4B-a,b). However, approximately 20% of Flag-Matrin-3-Ser208Ala-expressing cells expressed Matrin-3 in the cytosol (Fig. 4C-a). In addition, Flag-Matrin-3-Ser208Ala-transfected cells induced the extension of cellular processes (Fig. 4C-a). Process extended cells expressing mutant Matrin-3 (Ser208Ala) were merged with Tuj1^+^ cells (Fig. 4C-a,b). Tuj1^+^ cells were increased by 25% compared to cells transfected with Flag-Matrin-3 (Fig. 4C-b). Thus, phosphorylation of Matrin-3 at Ser208 could be essential for FGF2-dependent maintenance of the NSC system (Fig. 4D).

## Discussion

The mechanism of maintenance of the undifferentiated states of NSCs is poorly understood. Several studies have attempted to identify molecules that control NSC proliferation and differentiation. The present data reveal Matrin-3 as a novel key regulator of NSC differentiation via its expression and increased phosphorylation. The importance of Notch, Hes, Ascl 1, Neurod, Neurog, and DISC 1 in the regulation of differentiation of NSCs has been described^7,20,21^. Among them, basic helix-loop-helix **(**bHLH)-type transcription factor maintains the undifferentiated state of NSCs and, in particular, the Notch-Hes signal plays a major role in self-renewal^21^. Repressor-type bHLH transcription factors Hes1, Hes3, and Hes5 are expressed in NSCs during development and act as effectors of Notch signals to suppress neuronal differentiation^21,22^. Similar to Hes, Matrin-3 is a nuclear factor that maintains NSCs. Whether the signal transductions of Matrin-3 and the Hes family are common or independent is unknown.

As with Matrin-3, some DISC 1 localizes in the nucleus^23^ and regulates the differentiation control of NSCs by phosphorylation. DISC1 phosphorylation switches the development stage from prognostic cell division to nerve cell migration^7^. Moreover, although a DISC1 mutant was reported to impair Wnt/GSK3β signalling and brain development^24^, it is believed to be related to the neuropsychiatric phenotype. Matrin-3 mutations also result in familial amylotrophic lateral sclerosis^25^. As a result, dysfunction of NSC differentiation regulators and disruption of switching impair normal signal transduction and brain development, and may result in neurological disorders. In the future, we would like to clarify the detailed molecular mechanism of the influence of NSC differentiation regulating factor on the relationship between the breakdown of brain structure and function and the neuropsychiatric phenotype.

Matrin-3 is a 125-kDa nuclear protein^26,27^ that plays roles in chromatin organization, DNA replication, RNA processing, and homeodomain transcription program^28,29^. The current data demonstrate that Matrin-3 phosphorylation maintains NSCs. FGF2 induction maintains the undifferentiated states of stem cells, such as NSCs^30,31^. However, the underlying mechanism is unclear. In the current study, 2D-DIGE analysis demonstrated that the nuclear protein Matrin-3 was most phosphorylated upon FGF2 stimulation. We focused on Matrin-3 because it is the only protein that maintained the undifferentiated state of NSCs upon FGF2 stimulation in the 2D-DIGE analysis and because of the hyper-phosphorylation.

The 2D-DIGE method is a useful tool to reveal functional post-translational modifications of nuclear factors that cannot be detected with DNA microarray. A shotgun proteome approach using nano-liquid chromatography (nanoLC) tandem mass spectrometry incorporating phosphopeptide enrichment can detect phosphorylated peptides in a highly sensitive manner^32^. However, proteomics analysis using 2D-DIGE can clearly indicate the molecular weight and pH of proteins in 2D-electrophoresis, provides more credible results concerning nuclear protein identification, and displays better quantitative reproducibility. Furthermore, since phosphoproteins shift to the acidic side in isoelectric focusing, 2D-DIGE advantageously detects protein phosphorylation changes directly. In this study, an acidic shift of spots corresponding to phosphorylated Matrin-3 protein was obvious following FGF 2 stimulation in the 2D-DIGE analysis. This is the first description of this finding

*In vivo* analyses were done to focus on Matrin-3 phosphorylation at Ser208 by ATM. PhosphoMatrin-3 appeared at the early embryonic stage, and ATM expression started slightly earlier than Matrin-3 phosphorylation. Furthermore, phosphoMatrin-3 at Ser208 was strongly expressed in the VZ and hippocampal dentate gyrus where NSCs are enriched. *In vivo*, Matrin-3-siRNA induced disordered SVZ and VZ layers, accompanied by decreased prevalence of Ki67+ and nestin+ cells and increased numbers of NeuN+ cells. In NSC cultures, siRNA-mediated knockdown of Matrin-3 induced the extension of cellular processes, decreased the proliferation of NSCs, and reduced the number of neurospheres derived from NSCs. These findings indicated the neuronal differentiation of NSCs. Therefore, Matrin-3 could play a role in NSC maintenance, retention of the layer structure, and maintenance of NSCs in the SVZ/VZ layers.

Inhibition of ATM kinase in cultured NSCs induced the extension of cellular processes, promoted neuronal differentiation, decreased cell proliferation, and induced Matrin-3 dephosphorylation. Flag-Matrin-3-Ser208Ala-expressing neurospheres were slowly reduced. Phosphorylation of Ser208 of Matrin-3 by ATM is involved in modulating the cell cycle and DNA damage response^17^. ATM also reportedly mediates the response to DNA double-strand breaks in human neuron-like cells and human neurons derived from stem cells^33,34^. Moreover, ATM-short hairpin RNA induces neuronal differentiation of proliferating cells^33^. Therefore, we suggest that Matrin-3 phosphorylation by ATM maintains NSCs. Generally, Matrin-3 is localized in the nucleus. However, the inhibition of protein kinase A-dependent Matrin-3 phosphorylation induces the export of Matrin-3 from the nucleus to the cytoplasm^35^. The overexpression of the Ser208Ala mutant of Matrin-3 shifted the localization of the protein from the nucleus to the cytoplasm and the induction of neuronal differentiation. The reason why Ser208Ala mutation induced neuronal differentiation could be that ATM could not phosphorylate Ser 208 of Matrin-3 because Ser 208 was replaced with Ala. As a result, cells could not maintain their undifferentiated state and neuronal differentiation occurred. Additionally, we observed only 20% of Flag-Matrin3-S208A-expressing cells in the cytosol. We suggest that the dominant negative effect occurred in native Matrin-3. These results indicate that Matrin-3 functions in the NSC nucleus.

Why is Matrin-3 phosphorylated by FGF 2 and ATM, and how does this maintain NSCs? In situations where FGF2 drives stem cell self-renewal, reactive oxygen species (ROS) are produced via the AKT and MEK signalling pathways^36^. On the other hand, ROS control by ATM is necessary for the maintenance of undifferentiated states^37^. When ATM-ROS control fails, stem cell aging is induced^37^. These facts support the suggestion that the regulation of ATM-ROS is activated by FGF2 stimulation, and the maintenance of undifferentiated states is also activated. Therefore, phosphorylation of Matrin-3 might contribute to stem cell maintenance by both FGF2 and ATM.

Concerning the influence of FGF2 and ATM on the localization of Matrin-3, it is highly likely that FGF2 does not directly affect the altered localization of Matrin3. Further, direct phosphorylation of Matrin-3 by ATM in the nucleus may be necessary, as evidenced by the altered localization of Matrin-3 by ATM inhibition.

In this study, 2D-DIGE demonstrated a role of Matrin-3 phosphorylation. Kinase-specific phosphorylation site prediction by KinasePhos 2.0 revealed five theoretical sites other than Ser208 that might be phosphorylated by ATM in Matrin-3. Moreover, based on the possibility that Matrin-3 is phosphorylated by kinases other than ATM, other phosphorylation sites may be involved in the maintenance of NSCs.

In conclusion, phosphoproteomics identified a novel and critical nuclear molecule, Matrin-3, that is responsible for the induction of NSCs by FGF2. *In vivo* and *in vitro* functional analyses demonstrated that Matrin-3 phosphorylation is essential for FGF2-dependent maintenance of NSCs. Matrin-3 provides new insights into the control of NSC cell fate by post-translational modifications.

## Materials and Methods

### Detection of phosphorylated proteins using 2D-DIGE

We applied 2D-DIGE and discovered Matrin-3 using this method (Fig. 1A).

Institute for Cancer Research (ICR) mice were obtained from Japan SLC, Inc. (Shizuoka, Japan). NSCs were isolated from telencephalons of mouse embryos (E14) (N = 80) as previously described^38^, and were cultured for 5 days to expand the NSC pool in N2-DMEM/F12 medium (Life Technologies, Inc., Carlsbad, CA, USA) containing 10 ng/mL human recombinant FGF2 (PeproTech, Inc., Rocky Hill, NJ, USA)^39^.

NSCs were stimulated with FGF2 for 0 or 1 h after a 6-h FGF2 deprivation. This FGF2 stimulation experiment was performed four times. The NSCs were harvested and fractionated to enrich nuclear proteins using a nuclear protein dissociation kit (Thermo Scientific Pierce, Rockford, IL, USA). Then, nuclear extracts were desalted using a 2D-Clean-up kit (GE Healthcare UK Ltd., Little Chalfont, UK) and dissolved in lysis buffer containing 8 M urea (GE Healthcare), 4% CHAPS (GE Healthcare), 0.5% DeStreak solution (GE Healthcare) and 30 mM Tris-HCl (pH 8.5). Each nuclear extract (internal control, 0 min and 1 h) (50 μg) were labelled with 400 pmol of different fluorescent dyes (CyDye DIGE Fluor Cy2, Cy3 and Cy5, minimal dye; GE Healthcare), mixed together, and separated on identical gels. The detailed CyDye-labelled protocols were previously described^40^.

Nonlinear, 24-cm, pH 3–11, immobilised pH gradient (IPG) gel strips (GE Healthcare) were used for isoelectric focusing (IEF). A total of 150 µg Cy-labeled protein in a final volume of 450 µL lysis buffer containing 8 M urea (GE Healthcare), 4% CHAPS (GE Healthcare), 1.2% DeStreak solution (GE Healthcare), and 0.5% IPG buffer 3–11 NL (GE Healthcare) was loaded onto the IPG gel strips, and was rehydrated in the dark at room temperature overnight.

IEF was performed using an Ettan IPGPhor system (GE Healthcare) under the following condition: held at 100 V for 2 h, held at 500 V for 1 h, ramped to 1000 V in 1 h, ramped to 8000 V in 3 h, and held at 8000 V for 8 h for pH 3–11 NL gel strips. For sample equilibration, the IPG gel strips were incubated with 10 mL of a solution containing 50 mM Tris-HCl (pH 6.8), 6 M urea, 2% (w/v) SDS, 0.002% bromophenol blue, 30% glycerol, and 100 mg DTT for 30 min and then with 10 mL of a solution containing 50 mM Tris-HCl (pH 6.8), 6 M urea, 2% (w/v) SDS, 0.002% bromophenol blue, 30% glycerol and 250 mg iodoacetamide for 15 min. Afterward, 150 µg of protein was applied to a 24-cm gel strip and separated by second-dimension electrophoresis using the Ettan DALTsix Electrophoresis system (GE Healthcare). A 12.5%, 257 mm W × 200 mm H × 10 mm T gel (Perfect NT Gel D, DRC, Tokyo, Japan) was used for SDS-PAGE.

The fluorescence intensities of Cy-labelled proteins were quantified by scanning the gels at each excitation wavelength (488 nm/520 nm for Cy2, 532 nm/580 nm for Cy3, and 633 nm/670 nm for Cy5) using a Typhoon 9400 laser scanner (GE Healthcare). A 2D-PAGE image for multiple samples was obtained from a single gel. Quantitative analysis of the protein patterns was performed using the image analysis software DeCyder (GE Healthcare).

### NanoLC-ESI-MS/MS Analysis

Selected protein spots were excised from a Deep Purple (GE Healthcare)-stained preparative gel, destained, and dried, followed by in-gel digestion with trypsin (0.1 mg/mL; Promega KK, Madison, WI, USA). The resulting peptides were extracted with 0.1% trifluoroacetic acid (TFA; Wako, Osaka, Japan)/50% acetonitrile (Wako), dried, and then reconstituted in 0.1% TFA. After desalting and concentrating with a Zip Tip microC18 (Merck, Darmstadt, Germany), the peptide samples were analyzed by nano-LC ESI-MS/MS using nano ESI-QqTOF mass spectrometer,QSTAR Elite/pulsar i DCQuad (AB SCIEX) equipped with a nano-flow RP liquid chromatography system, Ultimate 3000 (Thermo Scientific Dionex) as previously described^16^. Briefly, samples were loaded onto the nano-LC RP column, PepMap 75 um,150 mm, 3 um C18,100-Å (Thermo Scientific Dionex), with the flow rate of 200 nl/min, using a 120-min gradient of solvent A (2% ACN and 0.1% formic acid) to solvent B (85% ACN and 0.1% formic acid). MS data acquisition was performed using Analyst QS 1.1/2.1 software (AB SCIEX) with the scan cycles set to perform a 1 s MS scan followed by three MS/MS scans of the three most abundant peaks for 2 s each with 60 sec IDA mode. Identification of proteins and phosphorylation sites was performed using in house MASCOT (Matrix Science) by searching against the Uniprot or NCBInr database (taxonomy:Mus musculus)

### Protein identification

To identify the candidate proteins, data from NanoLC-ESI-MS/MS Analysis of proteins in 2D-DIGE were analysed using MASCOT software application 2.1.04 (Matrix Sciences).

The UniProt database (release-2010.03) and NCBInr (20060112) was used for the search. Number of entries in the database actually searched was 116042 (NCBInr Mus musculus (house mouse)).

The search parameters were as follows: Type of search, MS/MS Ion Search**;** taxonomy, Mus musculus, cleavage enzyme, trypsin; variable modifications, Carbamidomethyl (C), Oxidation (M), Phospho (ST), Phospho (Y); peptide mass tolerance of 0.3–0.8 Da, fragment mass tolerance of 0.3/0.5 Da, max missed cleavage, 1. Confident identification required a statistically significant (P < 0.05) protein score based on combined MS and MS/MS analysis. The experimental condition for protein identification was previously described^40^.

Data analyzed by Analyst QS 1.1/2.1 software is stored as a wiff file, and its Rawdata is submitted on MassiIVE (Accession No: MSV000080358).

### Statistical analyses

For comparing 3 or more groups, a one-way ANOVA followed by the Bonferroni/Dunn post-hoc test for multiple comparisons was applied. For comparison of 2 groups, Student’s t test was used. Probability values (*P* values) less than 0.05 were considered significant (*P < 0.05, **P < 0.01). The values depicted are the mean ± standard error of the mean (SE).

### Western Blotting

The samples were transferred onto a polyvinylidene fluoride membrane (GE Healthcare). The membranes were blocked with Tris-buffered saline (pH 7.4) containing 0.1% Tween 20 (Wako) and 5% skim milk, incubated with primary antibodies for 12 h, washed with Tween-TBS, incubated with HRP-conjugated secondary antibodies (GE Healthcare) against mouse or rabbit IgG for 1 h, and washed with Tween-TBS. Immunoreactivities to antibodies were visualised using an enhanced chemiluminescence system (PerkinElmer, Inc., Winter Street Waltham, MA, USA).

The following primary antibodies were used in this study: mouse anti-Matrin-3 (0.4–2.0 µg/mL, Lifespan Biosciences, Inc., Seattle, WA, USA); rabbit anti-phosphoMatrin-3 (pSer208) at 1:2000 (Bethyl Laboratories, Inc., Montgomery, TX, USA); mouse anti-β-actin at 1:10,000 (Sigma Aldrich, MO, USA), and mouse anti-ATM (2 µg/mL, Abcam, Cambridge, UK). The secondary antibodies were diluted to 0.1–1 µg/mL.

### Plasmids

The flag-Matrin-3 and flag-Matrin-3-S208A and -S208D plasmids used in this study were previously described^28^.

### *In Vitro* siRNA Knockdown

Cells at 70–90% confluency were prepared for transfection. A total of 2 μg of the plasmid encoding EGFP and mouse Matrin-3-siRNA (Santa Cruz Biotechnology, CA, USA) was cotransfected. Silencer Negative Control #1 siRNA (Life Technologies) was used as a negative control. The “no-siRNA” group was transfected with only the GFP plasmid, and the “negative control-siRNA” and the “Matrin-3-siRNA” groups were cotransfected with the GFP plasmid. After siRNA transfection, the NSCs were cultured for 24, 48, or 72 h.

### ATM Inhibitor Treatment

NSCs were incubated in culture medium containing 50 nM of an ATM inhibitor KU55933 (Merck) at 37 °C. To observe neuronal differentiation, ATM treatment was continued for 72 h. Stock solutions were prepared in DMSO.

### *In Utero* Electroporation and Histological Analyses

E14.5 ICR mice were used for in utero electroporation. The mixed-plasmid DNA solution was microinjected into the lateral ventricle of embryos in a pregnant mouse at E14.5. Matrin-3-siRNA or control-siRNA constructs were mixed with GFP plasmids and FAST-Green. GFP plasmids were adjusted to a final concentration of 1.5 μg/μL, and the Matrin-3-siRNA and control-siRNA constructs were adjusted to 50 μg/μL. An electrode was placed on the forebrain of each embryo and pulsed 6 times (1 time at 99 V for 10 ms, 5 times at 30 V for 50 ms) with an interval of 50 ms using an electroporator (CUY21EX, BEX, Tokyo, Japan). The embryos were dissected 72 h after electroporation at E17.5, and the brains were fixed and frozen. The frozen sections, 16-μm thick, were subjected to immunostaining for Ki67, nestin, and NeuN. If necessary, serial sections were prepared, and hematoxylin and eosin (HE) staining was performed to observe the layer structure of the cerebrum.

### Full image of Western Blotting and Compliance with Ethical Indicators

Full images for all 2D gels, 1D-WB and 2D-WB in the figures were shown in Fig. S8.

This study has been approved by Type 2 Genetically Modified Organisms Usage Safety Commission and Institutional Animal Care and Use Committee of Kumamoto University that the authors belong.

## Electronic supplementary material

Supplementary Information
