# Supplementary material for: Matrin-3 is essential for fibroblast growth factor 2-dependent maintenance of neural stem cells
Source: Sci Rep. 2018 Sep 7;8:13412. doi: 10.1038/s41598-018-31597-x (PMC6128890; doi:10.1038/s41598-018-31597-x)
Supplement: Supplementary file 1 — Supplementary Information [file 41598_2018_31597_MOESM1_ESM.pdf]

Supplementary Information

Title

Matrin-3 is essential for fibroblast growth factor 2-dependent maintenance of neural stem cells

Authors

Kanako Niimori-Kita[1*], Nobuaki Tamamaki[2], Daikai Koizumi[1] and Daisuke Niimori[3*]

Supplemental Experimental Procedures

2D-gel staining

To identify phosphorylated protein spots, the pool of nuclear protein extracts was separated by 2D-PAGE and stained with the ProQ Diamond phospho-protein stain (Life Technologies). 2D-gels were fixed with 50% methanol containing 10% acetic acid overnight, and washed 3 times in the deionized water for 15 min. Then, gels were stained with ProQ Diamond phospho-protein stain (Life Technologies) in the dark for 4 h, and washed with 20% acetonitrile containing 5% 1M sodium acetate (pH 4.0) 3 times for 1 h. Gels were washed with the deionized water for 5 min twice, and scanned with a Typhoon 9400 laser scanner.

To stain the whole protein spots, SYPRORuby gel staining was examined. Gels were fixed with 10% methanol containing 7.5% acetic acid for 30 min, and were stained with SYPRORuby protein gel stain (Life Technologies) overnight. Then, gels were washed with 10% methanol containing 7.5% acetic acid for 1 h and scanned with a Typhoon 9400 laser

scanner. Fluorescent gel images obtained from ProQ Diamond and SYPRORuby were merged with Cy-Dye labelled gel images on DeCyder software. For instance, using DeCyder software, protein spots were detected from total protein profiles (Cy-Dye staining) and 2D-phospho protein profile (Pro Q Diamond staining), respectively. Among them, protein spots commonly and highly expressed in each profile were selected. These selected protein spots were served as the landmarks. Then, both protein profiles were matched and compared by calculating the distance between the landmarks by "co-detection function" using DeCyder software proprietary algorithm. After spot matching and profiling, statistical quantitative analysis of the protein patterns was performed.

In vivo analyses

To prepare samples for western blotting, the cerebral tissues at the embryonic, postnatal, and adult stages were obtained from ICR mice.

To perform immunohistochemical analyses, the mice were anaesthetised with a lethal dose of intraperitoneal pentobarbital (150 mg/kg) and were perfused with PBS followed by 4% paraformaldehyde. The brains were fixed for 2 h on ice. After fixation, the brains were dehydrated in a 30% sucrose solution and embedded in OCT compound. The frozen brains were sectioned into 5-μm coronal slices. These slices were treated in a citrate buffer (pH 6.0) for antigen-retrieval in a microwave. Immunoreactive signals were detected using a tyramide signal amplification procedure (Life Technologies).

Cell transfection

NSCs were transfected with plasmids using electroporation (MicroPorator-mini, AR

BROWN, Tokyo, Japan) and/or FuGENE 6 transfection reagent (Promega). Transfection experiments were performed with 2–3 µg plasmid DNA in a final volume of 100 µL of OPTI-MEM I medium (Life Technologies). After the transfection media were removed, the media were replaced by NSC culture medium, and the cells were incubated for 72 h. These samples were used for immunostaining.

Immunofluorescence staining

NSCs were fixed with PBS containing 4% paraformaldehyde for 10 min. After washing with PBS 3 times, the NSCs were treated with PBS + 0.1% Tween 20 and then with 10% serum in PBST at room temperature for 30 min. The cells were incubated overnight with antibodies diluted in 10% serum in PBST. The immunoreactive signals were observed using an epifluorescent microscope system (Olympus, Tokyo, Japan).

The following primary antibodies were used in this study: goat anti-Matrin-3 (0.66 µg/mL, Santa Cruz Biotechnology); mouse anti-Matrin-3 (0.4–2.0 µg/mL, Lifespan Biosciences, Inc., Seattle, WA, USA); rabbit anti-phosphoMatrin-3 (pSer208) at 1:200 (Bethyl Laboratories, Inc., Montgomery, TX, USA); mouse anti- GFP at 1:2000 (Sigma); rabbit anti-GFP at 1:500 (MBL, Nagoya, Japan); mouse anti-Ki67 (0.5 µg/mL, BD Pharmingen, Franklin Lakes, NJ, USA); mouse anti-nestin (0.5 µg/mL, BD Pharmingen); mouse anti-Tuj1 at 1:2000 (Sigma); mouse anti-NeuN at 1:100 (Millipore, Billerica, MA, USA); mouse anti-flag at 1:300 (Sigma); and mouse anti-ATM (2 µg/mL, Abcam, Cambridge, UK). DAPI was used at 1:1000 (Sigma). DAPI was mixed with the mounting medium. The secondary antibodies were Alexa fluorescent dyes (Alexa594, Alexa568, and

Alexa488; Life Technologies) for each animal species and Cy5 (GE Healthcare). The secondary antibodies were diluted to 4–8 μg/mL.

Tissue sections of mouse and human hippocampus

Tissue sections of normal human adult brain hippocampus from an 82-year-old woman were obtained from US Biomax, Inc. (Rockville, MD, USA). Normal adult mouse brain tissue sections were isolated at postnatal day 30.

Neurosphere assay

NSCs were transfected with GFP plasmid/siRNA constructs or Flag-Matrin-3/-Matrin-3-S208A plasmids using electroporation (NEPA21, NEPA GENE Co., Ltd, Chiba, Japan) and were cultured for 48 h. Transfected NSCs (50,000 NSCs / dish) were detached and cultured with NSC culture medium containing 10 ng/mL FGF2 for 7 days under floating conditions using poly-HEME-coated 60-mm dishes. The number of GFP/Flag-positive neurospheres/dish was all measured. For observing neurospheres with KU55933 treatment, neurospheres were cultured with NSC culture medium containing 50 nM KU55933.

Supplementary Figures

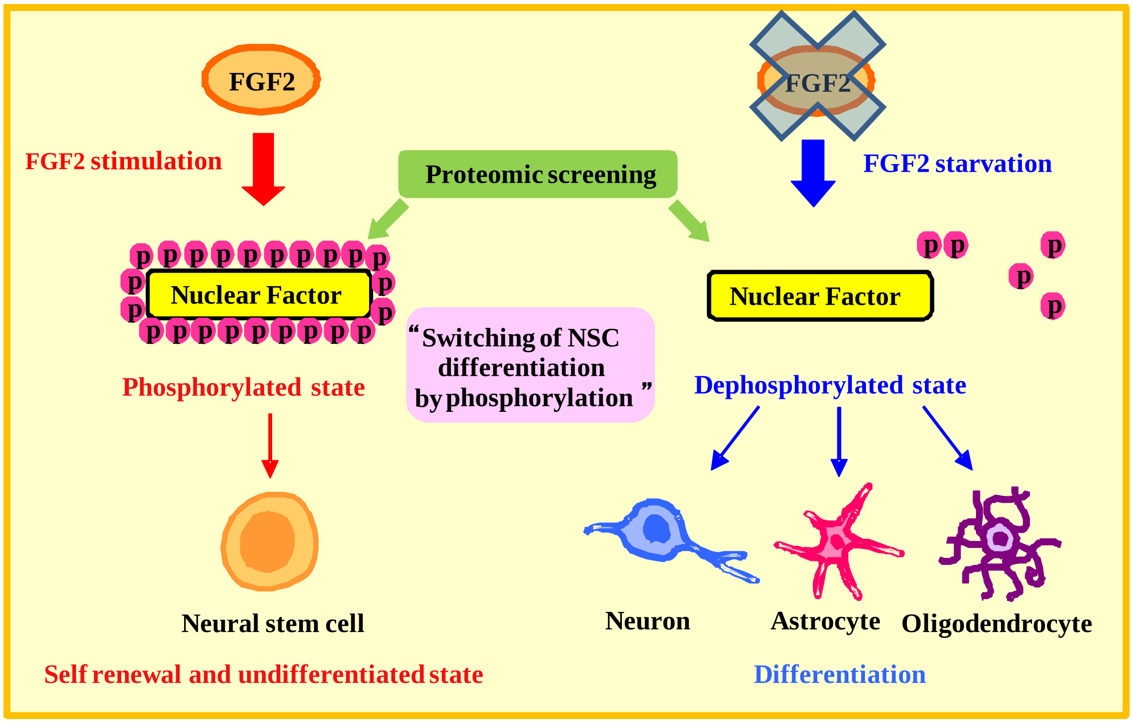

Fig. S1. Proteomics analysis of post-translational modifications of nuclear factors controlling NSC fate.

To identify proteins that regulate the switch from an undifferentiated to a differentiated NSC state, we searched for post-translational modifications (such as phosphorylation) of nuclear factors in response to FGF2 stimulation using proteomics analysis.

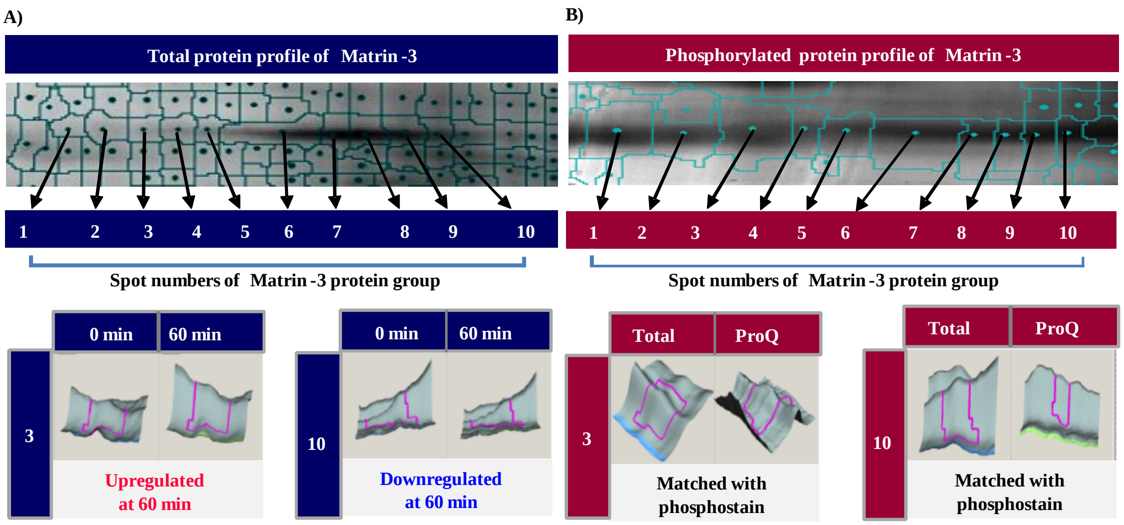

Fig. S2. Total and phosphorylated Matrin-3 protein profiles.

A, The total Matrin-3 protein profile. Lower panels: 3D images of spots 3 and 10. The numbers of spots 3 and 10 corresponded to those in the upper panel. 3D images of spots 3 and 10 (red-lined) show Matrin-3 expression, indicating that Matrin-3 expression levels increased or decreased following 1-h FGF2 restimulation. **B**, Protein profile of phosphorylated Matrin-3. Spots 1–10 were stained with ProQ Diamond. “Total” shows Matrin-3 expression on the master gel. “ProQ” shows phosphorylated Matrin-3. 3D image (red line) of “Matched with phosphostain” indicates that the Matrin-3-protein spot (left panel) is matched with the phosphoMatrin-3-protein spot (right panel). Spot 3 in the figure A and B corresponds to Spot 871 in the Fig.1B and Table.S1. Also, Spot 4, 5, and 7 correspond to Spot 872, 876, and 889 respectively.

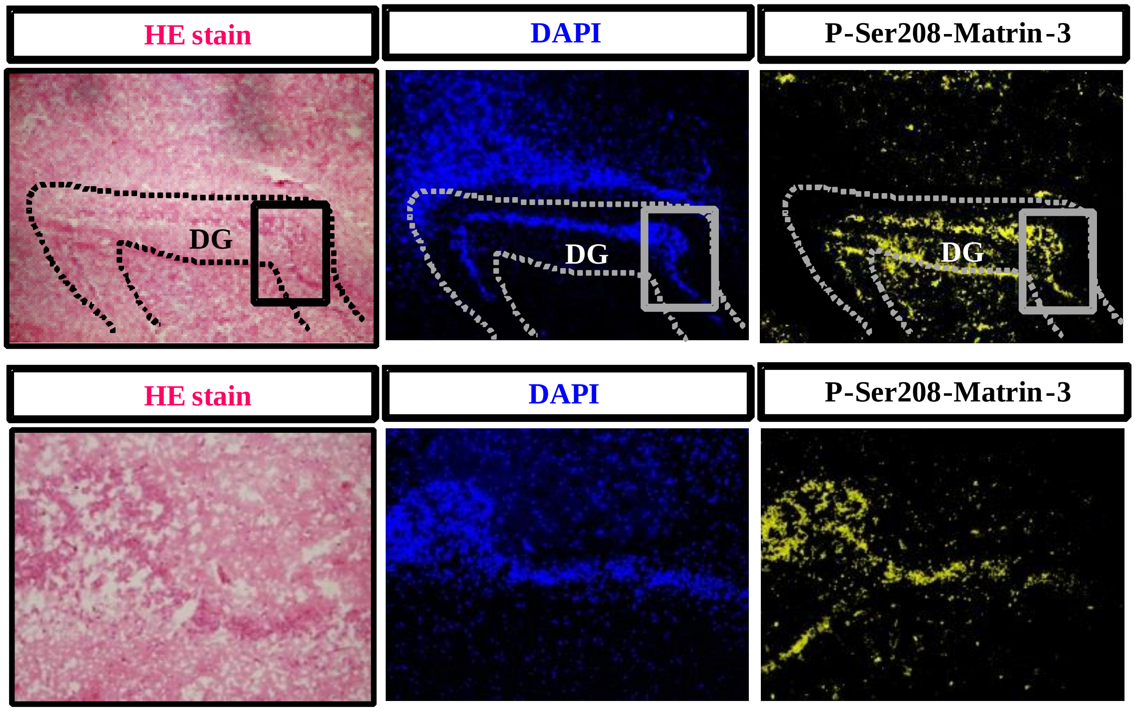

Fig. S3. Appearance of phospho-Matrin-3 (P-Ser208-Matrin3) in the human hippocampal dentete gyrus.

Appearance profiles of phospho-Matrin-3 (P-Ser208-Matrin3; yellow) in the human hippocampal dentete gyrus. A brain section from normal adult human frozen tissue was stained. The dotted line surrounds dentate gyrus area (DG). The bottom panel shows the enlarged area of the frame in the upper panel. HE staining revealed the dentete gyrus structure. DAPI was applied for the nuclear stain (blue).

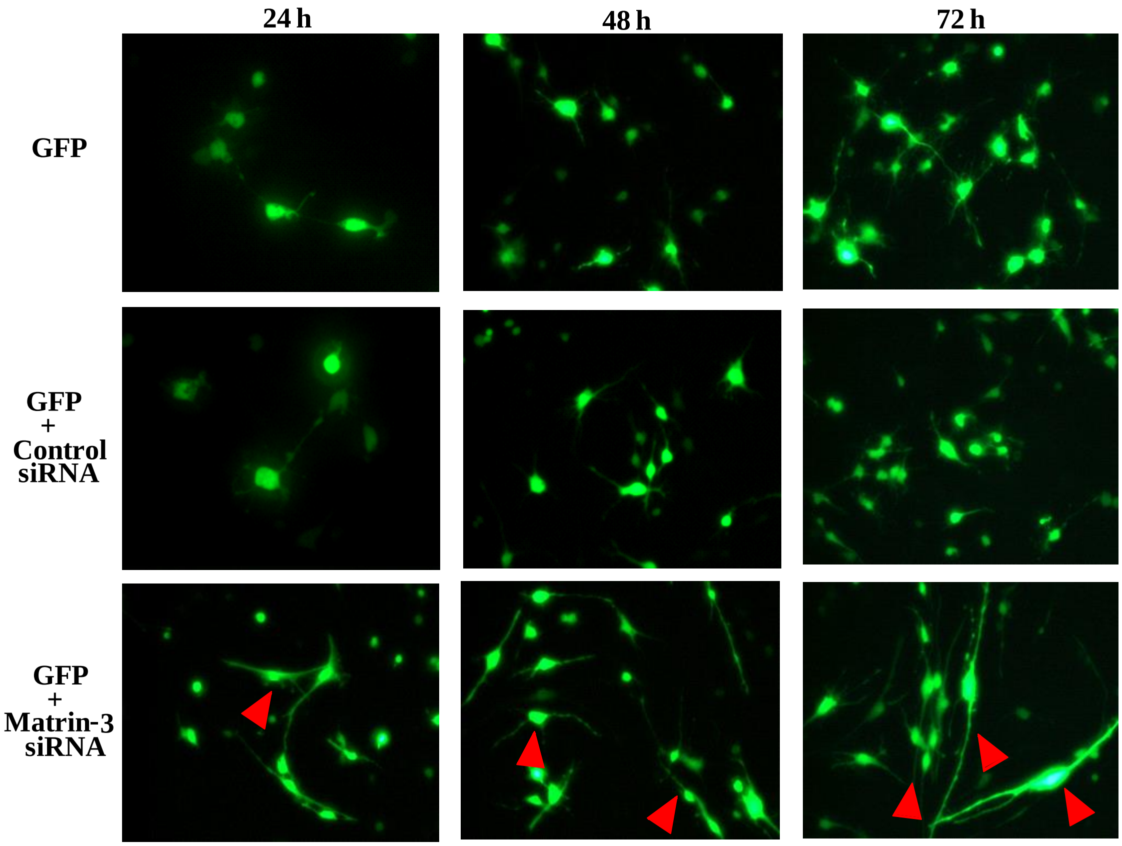

Fig. S4. Matrin-3-siRNA transfection triggers process extension in a time-dependent manner.

After siRNA transfection, NSCs were cultured for 24, 48, or 72 h. Morphological changes are observed after GFP staining. Red arrowheads indicate NSC process extension.

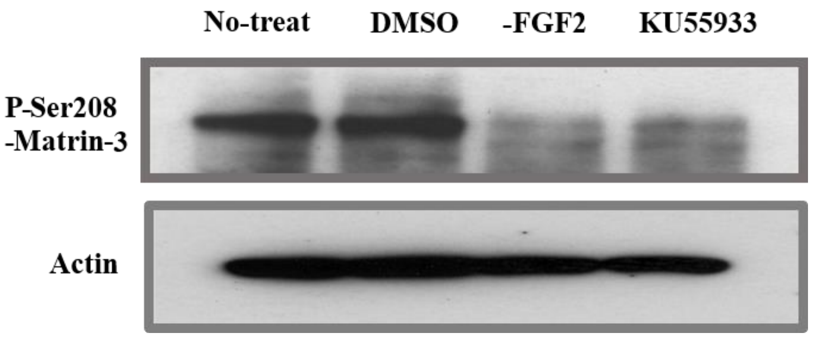

Fig. S5. Reduction of phospho-Matrin-3 (P-Ser208-Matrin3) in NSCs following treatment with KU55933.

The immunoreactive signals of phospho-Matrin-3 (P-Ser208-Matrin3) are detected. Actin are blotted as a control for protein expression. No-treat, no treatment; DMSO, treatment with DMSO only; –FGF2, FGF2 deprivation for 72 h.

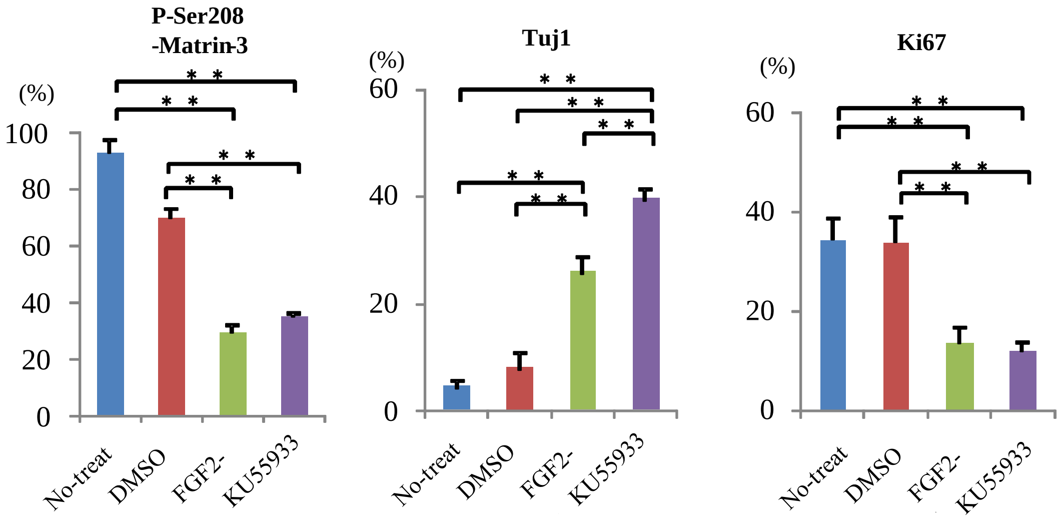

Fig. S6. Statistical measurement of the suppression of phospho-Matrin-3/Matrin-3 expression and neuronal differentiation by KU55933.

P-Ser208-Matrin-3$^+$, Tuj1$^+$, and Ki67$^+$ cells in DAPI$^+$ cells were counted. **$P < 0.01$ (one-way ANOVA plus Bonferroni/Dunn post-hoc test). Error bars, SE (5 separate experiments).

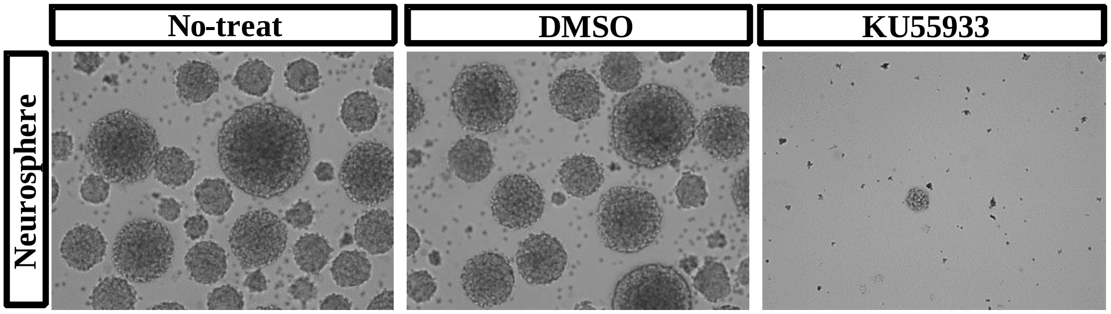

Fig.S7. KU55933 reduced the number of neurosphere-forming stem cells and those sizes.

Neurospheres were cultured with NSC culture medium containing 50 nM KU55933 for 7 days. Bar, 100 μm.

A)

Total protein profile

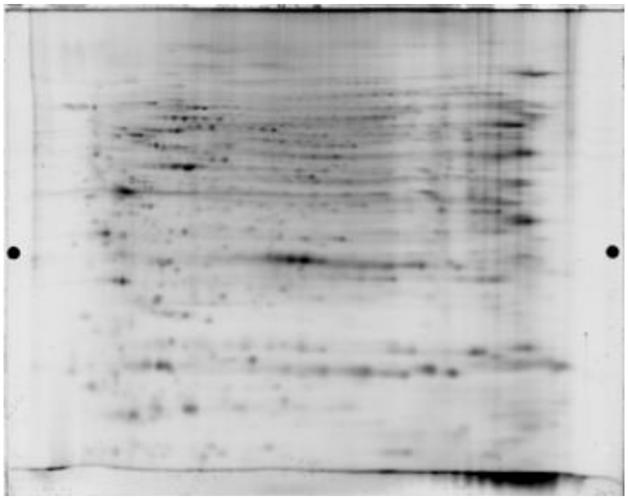

Phosphorylated

protein profile

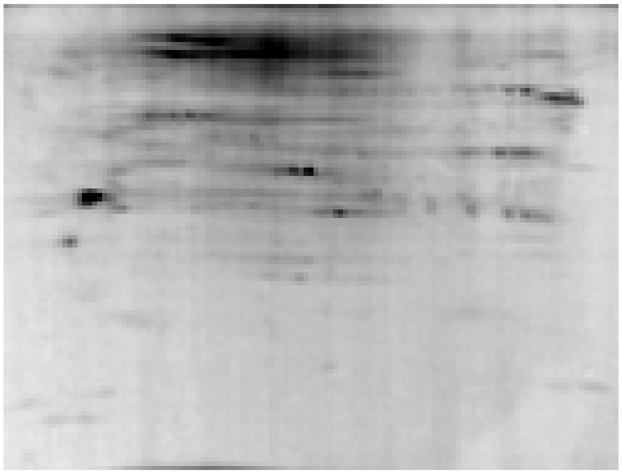

Cy-labeled

protein profile

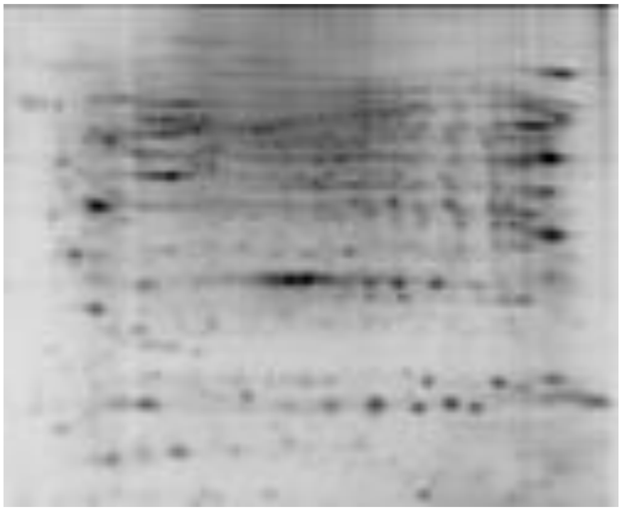

B)

Matrin-3

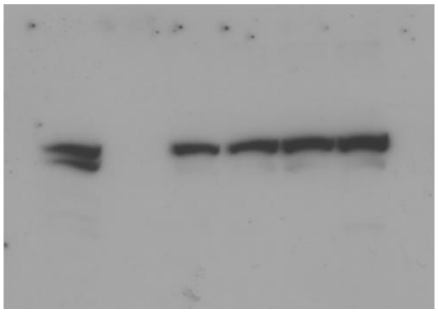

P-Ser208-Matrin-3

Actin

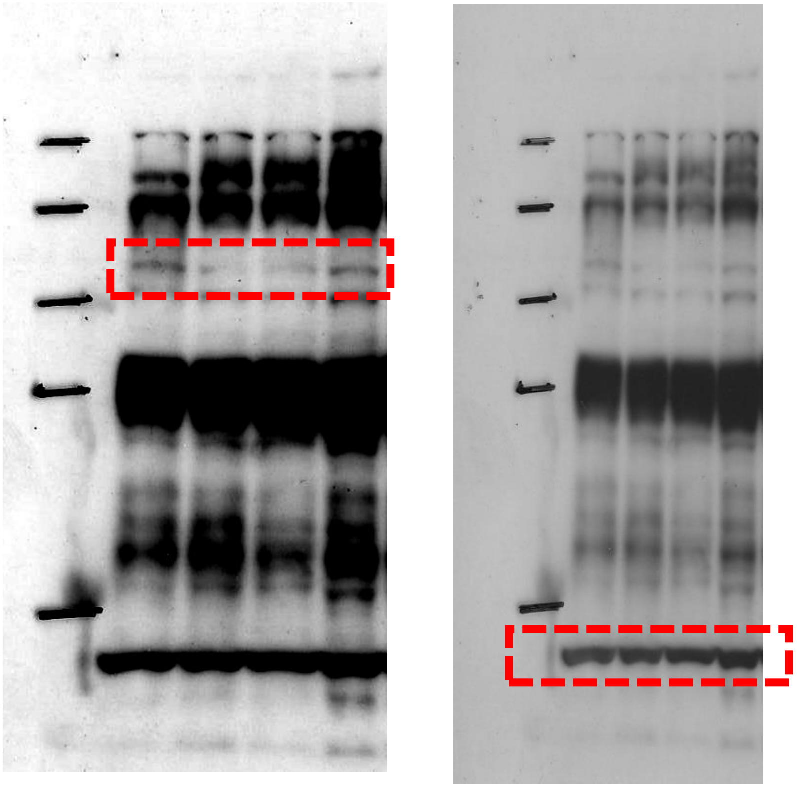

(C)

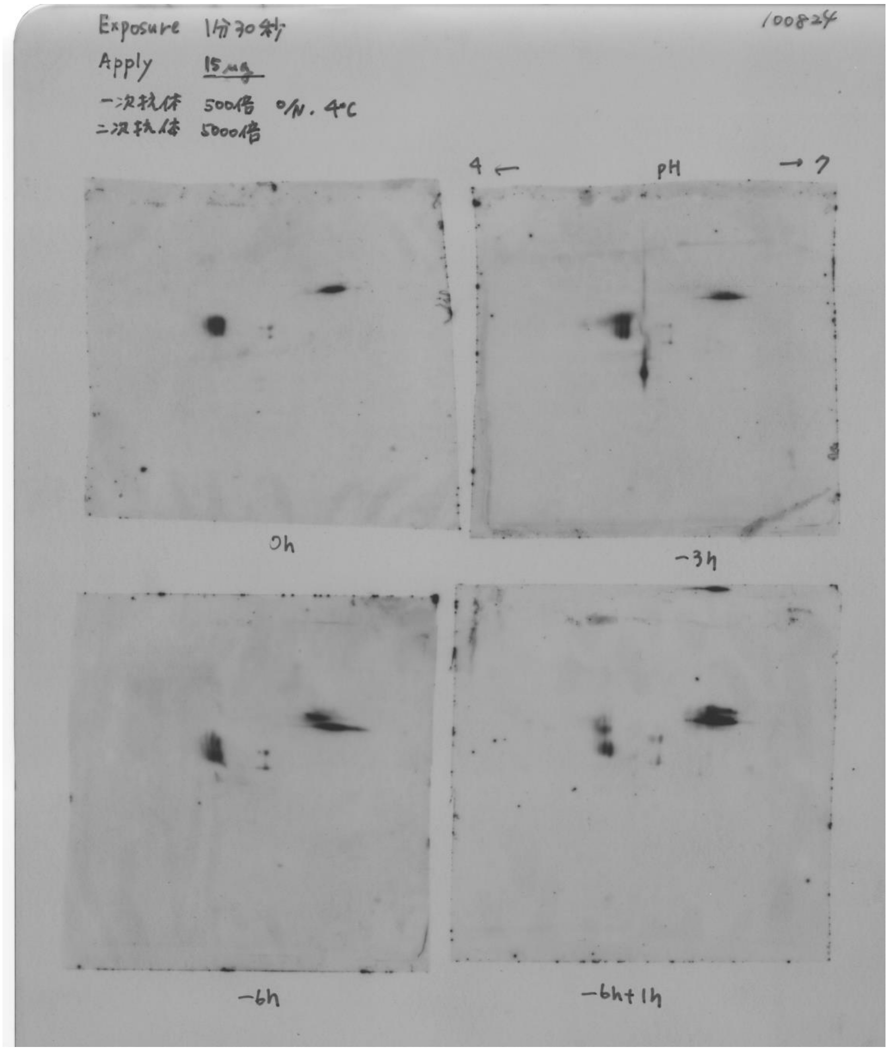
Exposure 1分30秒
Apply 15 μg
一次抗体 500倍 O/N. 4°C
二次抗体 5000倍
100824
4 ← pH → 7
0h
-3h
-6h
-6h+1h

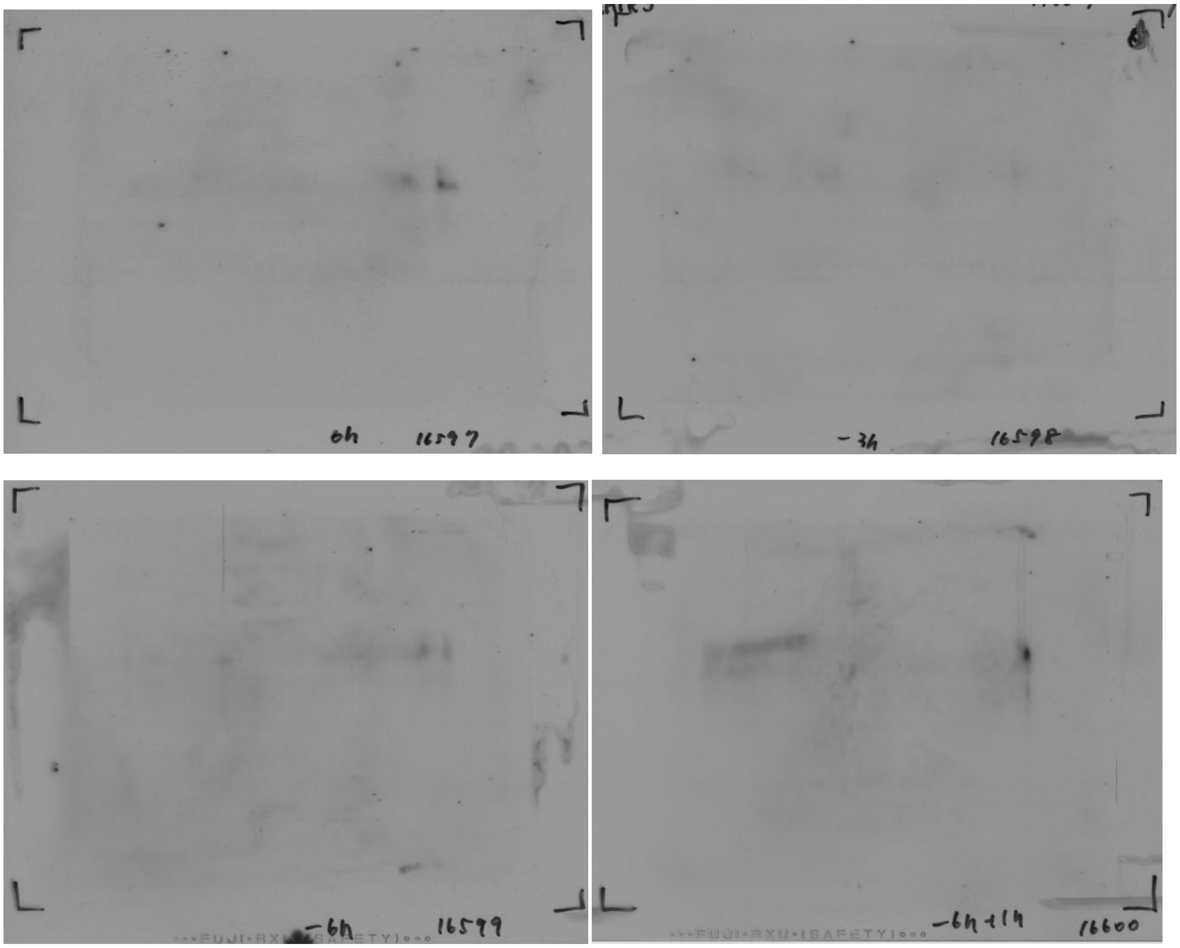
6h 16597
-3h 16598
-6h 16599
-6h+1h 16600

D)

Matrin-3

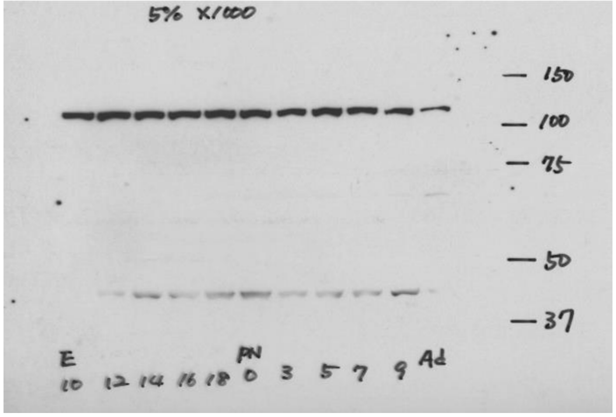

P-Ser208

-Matrin-3

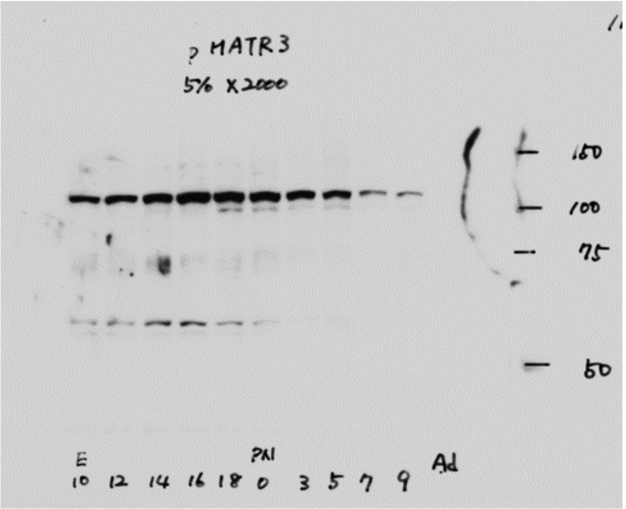

ATM

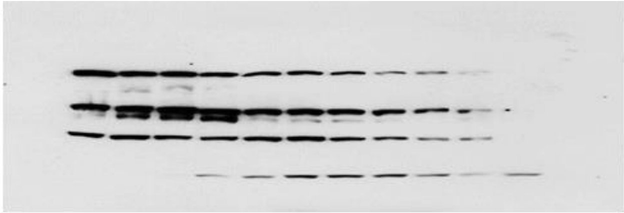

Actin

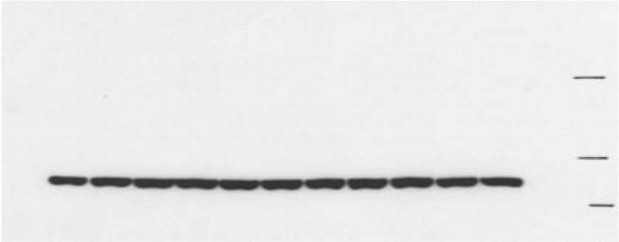

(E)

P-Ser208

-Matrin-3

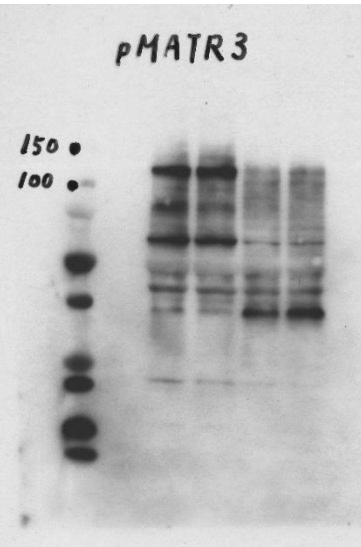

Actin

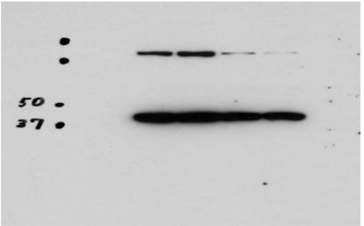

Fig.S8. Full images for all 2D gels, 1D-WBs and 2D-WBs shown in the figures.

A, Total protein-, phosphorylated protein- and Cy-labelled protein profiles. **B**, Full images of 1D-WB after the immuno-reaction using anti-Matrin-3, -phosphoMatrin-3 and -actin antibodies. **C,** Full images of 2D-WB after the immuno-reaction using anti-Matrin-3 and phosphoMatrin-3 antibodies. **D**, Full images of 1D-WB after the immuno-reaction using anti-Matrin-3, -phosphoMatrin-3, -ATM and -actin antibodies. **E**, Full images of 1D-WB after the immuno-reaction using anti-phosphoMatrin-3 and -actin antibodies.

These full images of 2D gels, 1D-WBs and 2D-WBs are corresponded to the figures of 1A, 1B, 2A, 2B, 2C, 2D, S2 and S5.

Spot No.	Protein Name	MW (Da)	pI	Accession No.	Protein Score	Sequence Coverage (%)	Number of peptides	ProQ Diamond Staining	Functional classification
871	Matrin-3	94752	5.87	Q8K310	374	24	9	Positive	Transcription regulation, DNA, RNA binding
872	Matrin-3	94752	5.87	Q8K310	487	25	14	Positive	Transcription regulation, DNA, RNA binding
876	Matrin-3	94752	5.87	Q8K310	379	23	10	Positive	Transcription regulation, DNA, RNA binding
889	Matrin-3	94752	5.87	Q8K310	654	31	15	Positive	Transcription regulation, DNA, RNA binding
889	olfactory receptor GA_x5J8B7TSHCU-6-512	18856	8.96	Q8K515	44	4	1	Positive	Olfactory receptor
889	cystinosis, nephropathic	42203	8.92	Q542U5	44	2	1	Positive	L-cystine transmembrane transportor
1481	Hydroxysteroid (17-beta) dehydrogenase 4	79432	8.76	P51660	59	3	2	Positive	Fatty acid metabolism
1742	Ubiqln1 protein	61937	4.86	Q8R317	276	11	8	Positive	Regulation of protein ubiqiotination
1848	Ulip3 protein	62142	6.39	P97427	103	57	21	Positive	axon guidance, invasive growth cell migration
1848	CCT (chaperonin containing TCP-1) zeta subunit	57968	6.63	P80317	615	34	12	Positive	'de novo' protein folding
1848	dihydropyrimidinase-related protein 4	61922	6.51	O35098	260	17	6	Positive	Neuron death Neuron projection guidance
1848	heterogeneous nuclear ribonucleoprotein L	60085	8.33	Q8R081	241	15	7	Positive	mRNA processing
1848	chaperonin subunit 6b (zeta)	58148	6.96	Q497N0	158	8	3	Positive	Protein folding Toxin transport
1848	NonO	54564	9.01	Q99K48	122	7	3	Positive	Circadian rhythm
1848	splicing factor U2AF	53483	9.19	P26369	73	19	3	Positive	mRNA splicing, via spliceosome
1848	TOAD-64	62239	5.95	P47942	71	3	2	Positive	Axon guidance
1848	Ulip	61897	6.04	Q62188	49	2	2	Positive	Actin crosslink formation
1848	dihydropyrimidinase	56725	6.74	Q9EQF5	46	4	2	Positive	Beta-alanine metabolic process
1932	Nucleosome assembly protein 1-like 4, isoform CRA_C	24807	5.11	Q78ZA7	49	2	1	Positive	Nucleosome assembly
2053	heterogeneous nuclear ribonucleoprotein K, (hnRNPK)	50976	5.39	P61979	284	25	11	Positive	mRNA processing mRNA splicing
2053	vimentin	53689	5.05	P20152	611	30	11	Positive	Astrocyte development
2053	p59 immunophilin	51540	51540	P30416	440	31	11	Positive	Androgen receptor signaling pathway
2053	tubulin, alpha 1	50104	4.94	P68370	288	27	8	Positive	Microtubule-based process
2053	Protein disulfide isomerase associated 3	56643	5.88	P27773	72	9	3	Positive	Cell redox homeostasis
2053	thymopoietin epsilon	45944	9.45	Q61029	72	7	2	Positive	Regulation of transcription, DNA-templated
2053	Desmin	53465	5.21	P31001	67	5	2	Positive	Intermediate filament organization
2053	peripherin	54235	5.4	P15331	62	4	2	Positive	Intermediate filament cytoskeleton organization
2053	3-hydroxy-3-methylglutaryl-Coenzyme A synthase 1	57516	5.65	Q8JZK9	59	4	2	Positive	Brain development
2390	Integrase interactor 1 (INI1)	44141	5.86	Q9Z0H3	545	32	10	Positive	Cell cycle Neurogenesis
2390	PTB-associated splicing factor	43810	9.45	Q8VIJ6	80	3	2	Positive	Alternative mRNA splicing, via spliceosome
2390	ARP1 actin-related protein 1 homolog B	42255	5.98	Q8R5C5	62	5	2	Positive	ATP binding
2904	Ubiquitin C-terminal hydrolase 37 (USP37)	110062	5.91	Q8C0R0	89	5	2	Negative	Ubiquitin dependant-protein catabolic process
3216	Methyl-CpG binding domain protein3 (MBD3)	20675	8.25	Q9Z2D8	335	21	5	Negative	Transcription regulation
3216	carnitine deficiency-associated protein CDV3A	24324	5.84	Q4VAA2	171	25	4	Negative	Cytoplasm, nucleolus
3957	ATP synthase, H+ transporting, mitochondrial F0 complex, subunit d	18784	5.52	Q9DCX2	283	48	6	Positive	ATP synthesis coupled protein transport
4861	Fatty acid binding protein 5, epidermal (FABP5)	15127	6.14	Q05816	72	39	4	Positive	glucose transport
4861	glial fibrillary acidic protein, astrocyte	49878	5.28	P03995	79	6	2	Positive	Astrocyte development
4861	Ribosomal protein S12	14505	6.82	P63324	46	8	1	Positive	Response to organonitrogen compound

Table. S1. Summary of the proteins altered in response to FGF2 stimulation and identified by mass spectrometry analysis.

The spot number corresponds to the annotation shown in Fig. 1B. The spot number list identified by mass spectrometry analysis shows the protein name, molecular weight (MW), isoelectric point (pI). The accession number indicates the number for UniprotKB, the protein research database. The protein scores indicate the ion scores as a non-probabilistic basis for ranking protein hits. Sequence coverage refers to the occupancy of the detected peptide sequence in full length. For all protein identifications, we indicated number of distinct peptides assigned for each protein. Positive or negative for ProQ Diamond indicates that the protein did or did not stain with ProQ Diamond. Functional classification indicates the protein function from previous reports. Accession numbers, theoretical MW/pI, were obtained from UniprotKB, and protein scores were obtained with Mascot MS/MS ion searches of the spectrum data by nanoLC-QQTOFMS/MS analysis using NCBInr mouse database. Theoretical MW/PI may be different from the measured value. Keratin is omitted from the list. Matrin-3 that we identified was 125 kDa, and the MW was corresponded to that reported in previous papers (24, 25).
